# Parenting in the Digital Age: A Scoping Review of Digital Early Childhood Parenting Interventions in Low- and Middle-Income Countries (LMIC)

**DOI:** 10.3389/phrs.2024.1607651

**Published:** 2025-01-21

**Authors:** Lena Jäggi, Stella M. Hartinger, Günther Fink, Dana C. McCoy, Milagros Alvarado Llatance, Kristen Hinckley, Lucero Ramirez-Varela, Leonel Aguilar, Andreana Castellanos, Daniel Mäusezahl

**Affiliations:** ^1^ University of Basel, Basel, Switzerland; ^2^ Department of Epidemiology and Public Health, Swiss Tropical and Public Health Institute, Allschwil, Switzerland; ^3^ School of Public Health and Administration, Universidad Peruana Cayetano Heredia, Lima, Peru; ^4^ Harvard Graduate School of Education, Cambridge, MA, United States; ^5^ Department of Computer Science, ETH Zurich, Zurich, Switzerland; ^6^ Afinidata, Guatemala City, Guatemala

**Keywords:** early child development, digital intervention, parenting, stimulation, app

## Abstract

**Objectives:**

This scoping review examines the evidence and knowledge gaps regarding the effectiveness of digital early childhood parenting interventions in Low- and Middle-Income Countries (LMICs).

**Methods:**

Using PRISMA-ScR and PICOS frameworks, we systematically reviewed studies published since 2010 from four databases, focusing on the impact of digital parenting interventions on Early Childhood Development and parent-level outcomes.

**Results:**

Of 1,399 studies identified, 13 met inclusion criteria, evaluating digital interventions for parents of children aged 0–5 years. These interventions included digital-only and hybrid approaches, leveraging technologies for tasks such as sharing health and ECD information, reminders, group chats, or screening. Among ECD studies, three of four with parent-reported outcomes found positive effects, but none of three using direct assessments did. Parent-level outcomes, such as mental health and parenting behaviors, showed consistent positive impacts.

**Conclusion:**

Digital parenting interventions are feasible in LMICs but face challenges in implementation and reaching vulnerable families. Most studies are small-scale with variable designs and outcomes. Rigorous, high-quality studies are needed to establish effectiveness and optimize implementation strategies before these programs are deployed at scale.

## Introduction

Skills acquired during early childhood, including language, cognition, and social-emotional abilities before age 5, are essential for later educational, emotional, and economic achievements [1]. Consequently, delays in these foundational Early Childhood Development (ECD) skills can significantly impact a child’s life trajectory. This emphasizes the importance of prioritizing strategies to support ECD, especially in Low- and Middle-Income Countries (LMIC), where it is estimated that almost 45% of children under 5 fail to reach their full developmental potential [2, 3]. Parenting interventions are gaining prominence as crucial strategies for enhancing ECD outcomes spanning diverse socioeconomic contexts, as documented in the Lancet series on ECD [4, 5]. Notably, parenting interventions in LMICs are especially impactful and exhibit over three times the effect on children’s cognitive, language, and motor development compared to those implemented in High-Income Countries (HICs) [6].

An expanding body of evidence underscores the increasing popularity of digital interventions to improve maternal and child health outcomes worldwide over the last decades [7, 8]. This trend can be attributed to the rapid global expansion of cell phone coverage and internet access, providing continual opportunities for health and educational systems to engage with families remotely [9–11], including vulnerable and hard-to-reach families in LMIC [7, 8]. Traditional ECD interventions are typically dependent on home visitations, face-to-face interactions, or community groups [6, 12]. However, they are expensive and can encounter disruptions due to unforeseen budget constraints, extreme events, or global occurrences such as COVID-19. This underscores the need for innovative solutions that allow remote delivery. Digital parenting interventions use technologies such as computers, apps, or (smart-) phones to enhance parenting skills and practices that promote ECD, including stimulation, early learning, and responsive parent-child interactions [13, 14]. In response, digital ECD interventions targeting parents of young children are increasingly implemented globally as potential tools to effectively bridge educational and service delivery gaps [11, 13, 14].

While some digital ECD parenting interventions show promise, the scarce data that exists on the effectiveness of such programs largely stems from high-income settings [13, 14]. Recent reviews highlight the potential of scalable and cost-effective digital interventions for maternal and child health in LMICs [1, 14] but little is known about the feasibility of using and adapting, and the compliance of digital ECD parenting interventions in LMICs.

Even more importantly, the impact of such digital ECD programs on child development and parental outcomes in these contexts remains unclear.

This scoping review aims to systematically assess and consolidate the current literature on digital interventions designed to enhance ECD in LMICs, focusing on those that are exclusively digital or include a significant digital component.

## Methods

### Search Strategy

We used the Preferred Reporting Items for Systematic Reviews and Meta-Analyses Extension for Scoping Reviews (PRISMA-ScR; Supplementary Appendix S1) and the Population, Intervention, Comparator, Outcome, and Study types (PICOS) framework to structure our review (Table 1). No separate protocol was published.

**TABLE 1:** Population, Intervention, Comparator, Outcome, and Study types (PICOS) framework (Allschwil, Switzerland. 2024).

Population	Parents or other primary caregivers of young children (0–5 years) in low- and Middle-income countries as listed by the world bank in 2019, compiled in the cochrane EPOC LMIC filter 2020
Intervention	Digital interventions for parents of young children (0–5 years), i.e., interventions focusing on responsive caregiving or opportunities for early learning, that are web-based, use an app, or use (smart)phones to deliver or augment the intervention (e.g., to personalize a curriculum or substantially expand the reach of the intervention), and have been published since 2010.
Comparator	No comparator was required
Outcome	Primary outcome was child development status or quality of parent-child interactions. Secondary outcomes included any outcome on the parent level, such as changes in parental knowledge, parenting beliefs, parenting self-efficacy and parental mental health.
Study types	Peer reviewed intervention studies including qualitative, quantitative, pilot and feasibility studies that evaluated at least one primary or secondary outcome.

After a preliminary literature review we consulted with a librarian to group a combination of MeSH terms, subject headings and text words in title and abstract to group the Boolean search into three thematic blocks: 1) young child terms (population) AND parenting/parent terms (population, content of intervention) AND child development terms (primary outcomes), AND 2) digital terms (type of intervention), AND 3) LMIC terms (Cochrane EPOC LMIC Filter 2020; location of population). We searched peer-reviewed articles in 4 databases (Pubmed/Medline, APA PsycInfo, Scopus, and Web of Science) on 08 June 2022, updated on 12 July 2023. The full search string for PubMed on Ovid and the final number of records returned are included in Supplementary Appendix S2. We used the Systematic Review Accelerator web app [15, 16] to conduct searches in different databases and deduplicate records and screened articles in the Rayyan web app [17].

### Selection Criteria

We considered all original, peer-reviewed studies of digital parenting interventions (i.e., computer or app-supported, or using (smart-) phones) published from the invention of smartphones in 2010 to July 2023. Interventions had to target primary caregivers (henceforth “parents”) of children 0–5 years of age. While interventions could start during pregnancy, we required that they continued beyond birth. Interventions needed to focus on supporting parenting competencies and practices that promote ECD, such as opportunities for increasing stimulation, early learning, or responsiveness in parent-child interactions. We included interventions for special populations, such as for parents of children with a disability or for parents with mental health issues. Interventions needed to report an ECD outcome (primary outcome) or at least one secondary outcome on the parent level, such as parental knowledge, parenting beliefs, parenting self-efficacy, parental mental health or quality of parent-child interactions. The primary ECD outcome could be assessed by any means (e.g., direct assessment, parent report, observation).

Included interventions could be entirely digital or include a significant complementary digital component. The latter had to be a separate component that augmented the primary intervention, such as meaningfully expanding or personalizing a curriculum or expanding the reach of the intervention. For example, we excluded studies if digital tools were used exclusively to enhance in-person delivery, such as using videos or other digital teaching materials in live teaching sessions.

Finally, the study had to take place in a LMIC country, as defined by the World Bank list of countries 2019 compiled in the Cochrane EPOC LMIC filter 2020.

### Exclusion Criteria

We excluded grey literature. Relevant reviews, meta-analyses, or study protocols were also excluded, but their reference lists were screened to identify potentially eligible studies. We further excluded school- or center-based interventions but included interventions in samples recruited from daycare centers that targeted parents and parenting behaviors at home. We also excluded digital interventions without a specific objective to promote ECD, such as reminders to get vaccinations, medical checkups or after-delivery care, interventions encouraging breastfeeding, or interventions targeting perinatal maternal depression without an explicit parenting or ECD component.

### Screening and Data Extraction

**FIGURE 1:**
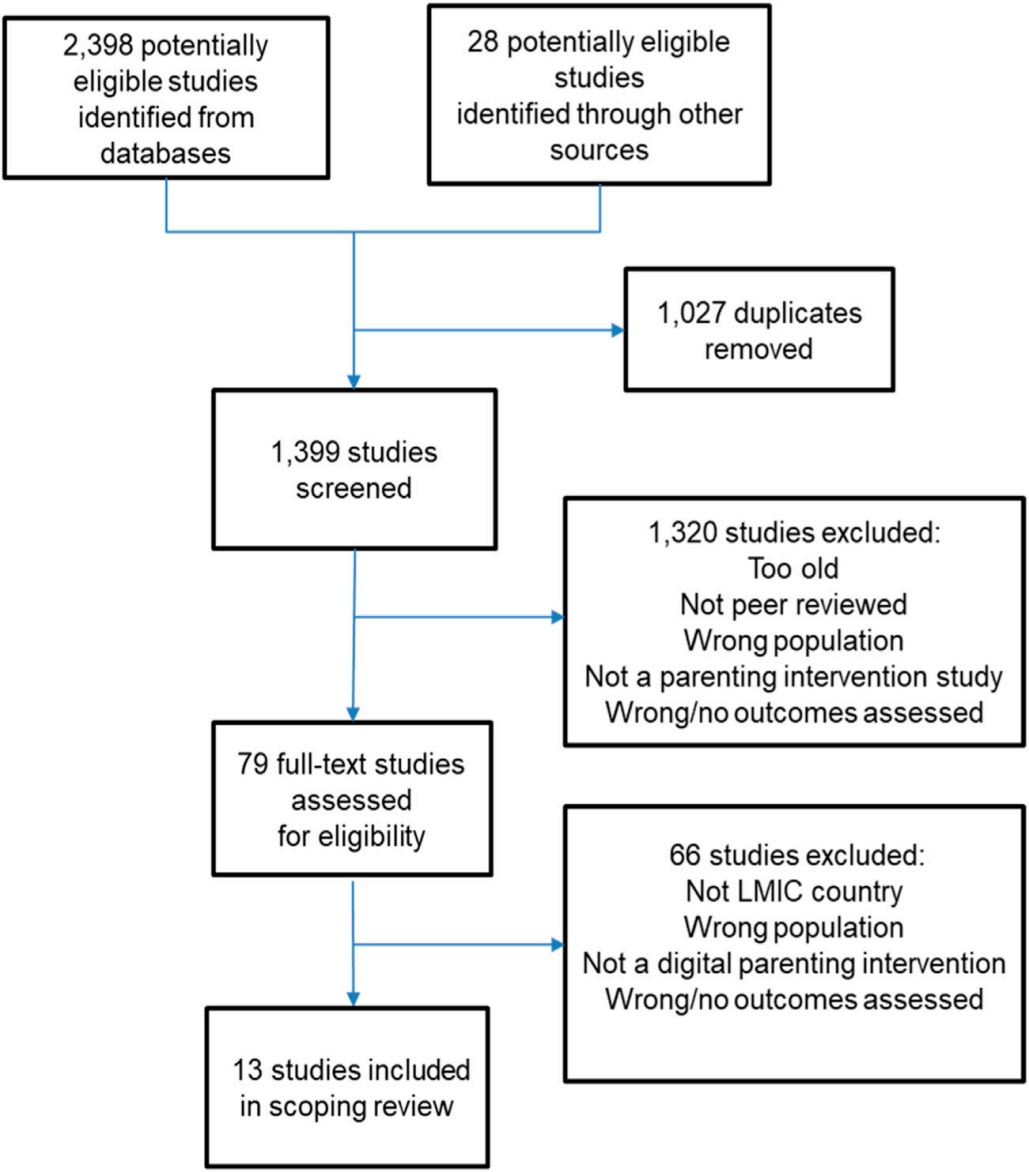
Screening flowchart (Allschwil, Switzerland. 2024).

After deduplication, articles were screened for relevance and the full text was evaluated. At least two of four reviewers (LJ, MA, KH, and LV) independently evaluated articles at each stage. Discrepancies in coding were resolved by consensus. Figure 1 shows a flowchart of the screening results. Extracted key data of the included studies are included in Table 2. A list of studies excluded during full-text screening and the reason for exclusion are included in Supplementary Appendix S3.

**TABLE 2:** Overview of included studies by type of intervention (Allschwil, Switzerland. 2024).

Authors	Year	Country	Study design	Sample size	Children’s age at baseline	Duration of intervention	Type of intervention	Description of intervention	Child outcomes^a^	Parent outcomes^a^
**Purely digital interventions**										
Balsa et al.	2021	Uruguay	Cluster RCT	N = 529 families	Mean 2 years	6 months	ECD parenting intervention	The Crianza Positiva intervention included 3 messages/week (text and audio format). Messages consisted of reminders, suggestions of action, and encouragement to reinforce and sustain positive parenting practices organized in 4 topics: attachment, protection, stimulation, and reflective function.	none	**Parent-child communication quality** (frequency and duration of vocalizations, turn-taking, adult response ratio, adult pitch range and average in 10-min video recording of free play) **Parental involvement in literacy activities with child**
Bloomfield et al.	2022	Uruguay	Cluster RCT	N = 529 families	Mean 2 years	6 months	ECD parenting intervention	The Crianza Positiva intervention included 3 messages/week (text and audio format). Messages consisted of reminders, suggestions of action, and encouragement to reinforce and sustain positive parenting practices organized in 4 topics: attachment, protection, stimulation, and reflective function.	none	**Parental time investment** (Frequency of involvement in activities with child) Availability of books and toys **Positive parenting**, (E2P) Violent discipline (MICS6) Time preferences (MCQ)Parenting stress (PSI/SF) Sense of competence (PSOC) Positive parenting knowledge
Huang et al.	2021	China	RCT pilot	N = 44 mothers	at birth	3 months, outcomes assessed at 6 months	Parenting and wellbeing intervention for first-time mothers	A customized intervention website with 5 components: learning, communication, ask-the-expert, baby home, and reminder forum. Participants logged on independently but received weekly reminder calls or Wechat messages to log on, if needed.	none	**Maternal self-efficacy** (SICS) **Postnatal depression** (EPDS) **Social support** (PSSS)
Le Roux et al.	2022	South Africa	RCT	N = 82 families	4–5 years	4 months	Early language development intervention for low-income families	The CareUp interactive smartphone application sent 3 push notifications/week with instructions and activities to help stimulate early language development at home. The App also provides parents with culturally appropriate resources, such as stories for shared book reading.	Language Development (ELLA; PPVT-4, TOPEL, and others)	none
Romski et al.	2021	South Africa	RCT pilot	N = 51 families	3–6 years	3 months	ECD intervention for parents of children with developmental language disorder	The self-guided app (“Nna le wena”) provides parents with sequential training on expressive communication strategies to use during routine activities (Bathing/Dressing, Book Reading, Mealtime, Play) each week, totaling 48 sessions. The intervention was an addition to monthly hospital-based speech therapy sessions for children.	Receptive and expressive language skills (MSEL subtests, SA-CPOLD)	none
Solís-Cordero et al.	2022	Brazil	RCT	N = 129 female caregivers	11–21 months	2 months	Parenting intervention to improve caregiver-child interaction for low-income families	The play-based BEM Program consists of 8 video classes and 40 text and audio messages on WhatsApp that teach caregivers how to play with children while doing typical household chores using materials available at home: The videos included information on safety, tips and benefits for caregiver-child interaction and child development.	General child development, **communication subscale** (ASQ-3, Brazilian version)	Quality of caregiver-child interaction**, intrusiveness subscale** (CIB) Engagement in age-appropriate play activities Sense of competence (PSOC) Perceived stress (PSS)
Trude et al.	2021	Brazil	Mixed-method pre-post pilot, no control group	N = 30 mothers	12–18 months	2 months	Parenting and social support intervention for mothers	3 WhatsApp-based maternal support groups, where moderators sent daily messages and weekly activities to encourage interaction and role modeling between participants, covering 4 main topics for 2 weeks each (child nutrition, child sleep, early learning and responsive caregiving, and maternal psychosocial wellbeing).	Food neophobia (FNS) Sleep behaviors (BISQ)	Nurturing care (mother–child interactions and early learning opportunities) **Postnatal depression** (EPDS) Self-efficacy Social support (SSQ)
**Combined Digital and In‐Person Interventions**										
Gureje et al.	2019	Nigeria	Cluster RCT	N = 686 pregnant women	unborn	Variable: 2 months antepartum, 2 months postpartum, then referral to additional services if needed based on maternal EPDS score	Mental health intervention with parenting component for mothers with peripartum depression	Stepped-care psychological intervention package including parenting skills information delivered by community midwives, who received supervision and specialist consultation via mobile phones. Mothers received mobile phone voice message appointment reminders and individualized therapy homework reminders.	Growth and health at 6 months (height, weight, history of illness, immunization) Motor and cognitive development at 12 months (BSID)	**Postnatal depression** (EPDS) Maternal Functioning and Disability **at 6 months** (WHODAS) Parenting skills (MAMA; HOME) **Exclusive breastfeeding**
Hamdani et al.	2015	Pakistan	Pre-post evaluation pilot, no control group	N = 68 families	2–9 years	6 months	Service delivery and parenting intervention for families with a child with developmental disorder	Part 1: Mobile phone–based interactive voice response system identified children with developmental disabilities. Part 2: Tablet-based ACT system to train community volunteers and then families on psycho-education and parenting skills. Themes included care for development, physical health, nutrition, parental stress, community participation, stigma and rights using “real-life” interactive narratives. Part 3: ACT system collects case management data to track child’s progress and aid in supervision and monitoring.	**Child Functioning and Disability** (WHODAS-Child), **Socioemotional Difficulties** (SDQ)	**Family stigma** (ISE Family version) **Family empowerment** (FES) Caregiver wellbeing (WHO-5 WBI)
Kumar et al.	2021	India	Mixed-methods program evaluation, with control group	N = 100 children	0–3 years	34 months	Set of maternal and child health interventions with various parenting components, including home visits, mother’s groups and outreach	Families and community health workers received daily SMS or phone calls related to ECD and parenting components (play and communication; feeding; prevention of injuries and diseases; timely recognition and treatment of illnesses, and, pregnancy). Providers were directed to include the “Message of the day” in their work, e.g., for discussion in home visits or weekly “voluntary mothers’ clubs” meetings and received an additional booklet with notes on each theme.	**Malnutrition** (weight for age, **stunting** and **wasting**)	none
Mwenda et al.	2023	Kenya	Randomized pretest-posttest (2 intervention arms), with posttest-only control group	N = 1011 adolescent mothers	0–2 months	9 months	Parenting and social support intervention for adolescent mothers	An interactive text messaging platform sent 5–10 messages/week on feeding, breastfeeding, immunization, general childcare and safety, developmental milestones, danger signs and when to seek care. Participants could also interact with experts through the platform and ask questions (limited intervention). Some mothers received additional weekly in-person social support groups (full intervention)	**General development** (DMC-III)	**Knowledge on childcare and development (**exclusive breastfeeding, immunization, feeding, and stimulation**)** **Exclusive breastfeeding**
Smith et al.	2023	Jamaica	RCT	N = 247 at-risk families	5–24 months	7–9 months	ECD parenting intervention for at-risk families	Digital adaptation of Reach Up home visit program. Parents received: parent manual and play materials; phone calls every 2 weeks to introduce language and play activities and review progress; and weekly text messages to reinforce the phone calls and provide encouragement.	none	Parent behaviors (adapted FCI) **activities with child**, **use of praise**, and availability of toys and picture books in the home
Westgard & Orrego-Ferreyros	2022	Peru	Mixed methods implementation evaluation, with control group	N = 186 families	not reported	16 months	ECD home visit intervention for low-income families	The tablet-based CHEST App supports home visitors with age-appropriate animated videos and health messages during home visits. The app supports case management, displays the health status of the child and next scheduled home visit, and uploads the data collected during the home visit to a server to facilitate supervision and monitoring.	none	**Knowledge on childcare and development (**nutrition, sanitation, hygiene, disease prevention and ECD)

Note:

^a^ significant positive intervention impact marked in bold.

ACT, Avatar-assisted Cascade; ASQ-3, Ages and Stages-3; BISQ, brief screening questionnaire; BSID, Bayley’s Scale for Infant Development; CIB, coding interactive behavior; DMC-III, developmental milestones checklist; E2P, positive parenting scale subscale; ECD, early child development; ELLA, language subtests of emergent literacy and language assessment protocol; EPDS, edinburgh postnatal depression scale; FCI, Family Care Indicators.; FES, family empowerment scale; FNS, food neophobia scale; HOME, inventory for measurement of the environment, Infant Toddler version; ISE , inventory of stigmatizing experiences; MAMA, maternal adjustment and maternal attitudes questionnaire; MCQ, monetary choice questionnaire; MSEL, mullen scales of early learning; PPVT- 4, Peabody Picture Vocabulary Test; PSI/SF, parental stress index; PSOC, parental sense of competence scale; PSS, perceived stress scale; PSSS, postpartum social support scale; SA-CPOLD, Caregiver-reported child communication success and difficulty; SDQ, strength and difficulties questionnaire; SICS, Self-efficacy in Infant Care Scale; SSQ, social support questionnaire; TOPEL, word definitions from test of preschool early literacy; Training RCT, randomized controlled trial; WHO-5 WBI, The World Health Organization- Five Wellbeing Index; WHODAS, world health organization disability assessment scale.

The data extraction table was developed based on the research questions and the PICOS framework and included basic demographic information about the sample (i.e., sample size, age), study design (e.g., pilot study, randomized controlled trial), country, general information on the intervention, information on the digital component of the intervention, and child development and parenting outcomes assessed. We conducted a descriptive analysis and summarized the strengths and weaknesses of research about digital ECD parenting interventions in LMICs.

## Results

In total, 1,399 potentially relevant studies were identified (Figure 1). The abstract and full-text screening resulted in 13 included articles covering 5 interventions from Central and South America [Jamaica, Uruguay (2 studies from 1 intervention), Brazil and Peru], 4 from Africa [Kenya, Nigeria and South Africa (2 studies)], and 3 from Asia (Pakistan, China, and India). See Table 2 for details.

### Study Characteristics

Among the 13 studies, 3 were cluster-randomized controlled trials (cRCT), 5 RCTs, 1 randomized study with posttest-only control, 2 studies without control groups, and 2 studies included control groups but did not provide information on randomization. The studies included 5 pilot or feasibility studies and 3 studies included qualitative components. The sample sizes included in the studies ranged from 30 to over 1′000, but 5 studies had fewer than 100 participants.

Most studies compared either a digital [18–23] or mixed intervention [24, 25] to no-intervention or usual-care control. In two studies [18, 19], all eligible families had completed an 8-week parenting workshop in-person before they were randomized to receive the purely digital intervention versus not. No studies directly compared a digital versus an in-person parenting intervention on ECD or parenting outcomes.

### Intervention Characteristics

As shown in Table 2, 6 of the interventions (7 studies) were only digital, while 6 interventions combined digital and in-person components. Interventions targeted different age brackets of children, ranging from starting before birth [26], at birth [20, 27], to spanning 2–9 years [28]. However, 9 of the studies targeted infants through children up to 3 years and only 3 studies included children who were older than 3 years. Purely digital interventions were comparatively shorter than mixed interventions and lasted between 2 and 6 months. In terms of the target population, the purely digital interventions consisted predominantly (5/6) of general parenting programs for healthy parents of typically developing children, though some targeted low-income families or first-time mothers. One exception was a parenting app that promoted language development at home for parents of children with a developmental language disorder who were already receiving the local usual care treatment of hospital-based speech therapy [22]. Among the mixed-delivery interventions, there were 4 general parenting interventions: one digitally supported home-visit intervention [29] a remote adaptation of a home visit intervention [25], a digital parenting and social support intervention for adolescent mothers [27] and a comprehensive set of general maternal and child health interventions [24]. The other 2 mixed interventions targeted special groups, namely, mothers suffering from peri-partum depression [26] and families of children with a developmental disability [28].

Most purely digital interventions delivered content via pre-programmed scheduled Whatsapp [18, 19, 23, 30], SMS [18, 19] or in-App push-notification [21] messages to parents. Similarly the limited intervention arm in Mwenda et al.’s study received scheduled messages on an unnamed interactive messaging platform [27]. Only one study used moderators in the Whatsapp groups to encourage and facilitate spontaneous exchange between participants, though these exchanges were organized around pre-defined bi-weekly topics [30]. Moving beyond using a digital way to deliver fixed content at specific time points, one intervention used a self-guided, manualized parent training program in an App [22] and one intervention used a self-guided web-based content program [20]. However, both self-guided interventions were in fact accompanied and used strategies to increase compliance of the participants: Huang and colleagues’ monitored log-on data and sent individual WeChat reminders to log into the website to participants who fell under a weekly minimum [20]. Parents in the intervention by Romski and colleagues [22] received the tablet on which the app was installed. They were instructed on how to use the app, and how to integrate the communication strategies described in the app into everyday activities with their children. Moreover, participants were asked to fill out weekly in-app questionnaires and brought the tablet in for study staff to download monthly usage data.

Mixed digital and in-person interventions varied in the importance of the digital component concerning the overall intervention. For example, in the Kenyan program for adolescent mothers, the digital component was the main part of the intervention, which was compared to an enhanced version with an additional supportive in-person element [27]. One study replaced in-person home visits with remote delivery of content via phone calls and smartphone messages due to the restrictions brought by the COVID pandemic and was thus not intentionally designed as a mixed intervention [25]. The other 4 mixed interventions were predominantly delivered in-person, with some enhancing digital components to support community health workers providing the intervention [29] or both the intervention providers and the participating families [24, 26, 28], making them more digitally-enhanced rather than true mixed-delivery interventions. The support for providers consisted mainly of facilitating scheduling, monitoring participation, providing access to supervision, tracking of ECD, or providing them with digital or interactive content to support their in-person training of families. On the other hand, families received text reminders of therapy homework or appointments [26], informational messages on maternal and child health and ECD [24], or computer-assisted disability screening via phone to connect them to a comprehensive set of interventions and services if needed [28].

The level of detail in the description of intervention content varied greatly, but most digital general parenting interventions delivered content that was intended to give parents concrete ideas for activities or parenting strategies centered around positive parenting and nurturing care, and/or early learning and language development, sometimes combined with general informational content on ECD. Three studies did not describe details of the parenting or ECD content [20, 24, 26]. Three interventions explicitly mentioned being based on the multi-lateral Nurturing Care Framework [23, 27, 30], 2 studies referenced the Crianza Positiva positive parenting program [18, 19] and one intervention was a new locally developed home visit curriculum [29]. Three interventions were digital adaptations of existing interventions: one study was an adaptation of the validated Wordworks early literacy program [21], Smith and colleagues [25] described the remote delivery of the Reach Up home visit curriculum, and Romski and colleagues [22] adapted a protocol for young children with developmental disorders. Finally, Hamdani and colleagues [28] developed their intervention based on the mental health gap intervention guide from the WHO.

### Intervention Compliance

In general, one important determinant of intervention success is participant compliance and participation. For the present sample of programs, all purely digital intervention studies and two mixed-delivery studies [24, 29] tracked program engagement with the digital component.

One study presented detailed results on program engagement and noted significant declines in participant contributions throughout their Whatsapp-based intervention [30]. Another study concluded they did not find significant impacts on child outcomes in their self-directed app intervention because most parents used the smartphone app very little; only 28.6% of parents in the experimental group used the application for more than half of the intervention period [21]. However, follow-up analyses did not find associations between app-use and child outcomes in their small sample size of 42 experimental families. Similarly, the only other study that looked at the influence of program engagement found no moderating effect of adherence on any of the child or parent outcomes [23]. In another intervention study in South Africa using a self-guided app, participation rates were much higher, and over half of the parents (13/20) completed almost all of the sessions [22]. However, this intervention was delivered via a tablet that was given to the parents at the beginning of the study, and was connected to monthly therapy visits to the hospital for the children, where parents met with research staff during their waiting time and could address any questions, clarifications, or comments about the intervention, including technical support.

Several other studies also used specific strategies to increase engagement with the digital intervention: One study [20] monitored logins and sent weekly reminders to mothers who did not use the program website at least twice weekly or who stayed logged on for less than an hour per week. Another intervention [18, 19] re-contacted intervention families and the childcare centers to get updated phone information and maximize the number of families who would receive the content on their phones. Finally, one study used research assistant participants to keep conversations flowing in some of their moderated Whatsapp groups, though this did not seem to make a difference in the overall contribution rates of the groups [30].

### Child Outcome Findings

Nine of the studies measured at least one child outcome: only 3 studies used a validated direct assessment of ECD [21, 22, 26], and 4 studies used validated parent-reported child development or child disability questionnaires [22, 23, 27, 28]. Two studies assessed sleep behaviors or malnutrition.

None of the 3 studies using direct assessments found effects on ECD, while 3/4 studies assessing ECD with validated parent-reported instruments found positive child effects. One study [27] found improved general ECD, while another study [23] found positive effects on language but not on socio-emotional, cognitive or motor development. Hamdani and colleagues [28] found decreases in the children’s general disability and socioemotional difficulties over time. Kumar and colleagues [24] found positive effects on malnutrition outcomes. One pre-post study found negative effects on child’s food neophobia over time [30]. No change in communication difficulty was found in another small study [22].

### Parent Outcome Findings

In 2 studies, direct measurements of parent-child interactions were conducted using video recordings [18, 23]. In both studies, the digital intervention group showed improved outcomes, with less intrusiveness in the caregiver-child interaction [23] and better communication quality and responsiveness [18].

Ten studies assessed self-reported parenting outcomes, such as parenting behaviors, skills or knowledge centered on positive parenting, nurturing care, and healthy child development; often using multiple measures. Most studies observed positive effects on self-reported parenting behaviors or parenting knowledge, such as increased involvement in games or literacy activities [18, 19, 25], increased use of positive parenting practices [25], increased exclusive breastfeeding [26, 27], increases in parenting self-efficacy [20], or more knowledge on child development [27, 29]. However, several studies found no change in some parenting outcomes, such as sense of parenting competence [23], parental knowledge [19], nurturing care behaviors [30], self-efficacy [19, 30], or parenting skills [26]. One study assessed stimulation, immunization and feeding parenting practices, but no results were reported [27]. In summary, all studies who assessed parent outcomes, found improvement on some parenting measure in their intervention groups, though not on all measures.

Six studies also assessed parent outcomes which were not directly parenting related, such as parental stress [19, 23], wellbeing [28], social support [20, 30], or depression [20, 26, 30]. All 3 interventions targeting maternal depression found lower rates of maternal depression [20, 26, 30], and one of the studies also found increases in social support [20]. However, the other 3 studies found no differences in parental wellbeing [28] or stress levels [19, 23].

## Discussion

This scoping review aimed to evaluate existing evidence on digital parenting interventions in LMICs. Among the 12 interventions covered in 13 studies meeting inclusion criteria, 6 were purely digital, while the remaining 6 combined digital and in-person components.

The great diversity of intervention content, delivery, targeted participants, duration, study design, and outcome assessments makes a comparison and summary of trends challenging. Furthermore, even though parenting interventions ultimately seek to improve ECD, only 6/13 studies included assessments of ECD. Finally, 5 studies had fewer than 100 participants and only 3 had sample sizes of over 500 participants, highlighting the early stage of the current state of the literature.

### Leveraging the Digital Component

Broadly, current programs can be divided into purely digital, digitally supported, and true mixed digital and in-person interventions. Concerning the second type, supporting known in-person interventions with a digital component showed promising results [24, 28, 29], both to make content more attractive for parents and to assist in efficient case management and quality control. For example, digital supports including innovative screening approaches, cascade training, and monitoring using a supervision system between trained agents have been used successfully to increase the reach and quality of interventions in the included studies.

In many studies of purely digital interventions [18, 19, 21–29], the digital component primarily consisted of changing the delivery method from in-person to remote or digital. Specifically, most of these programs mirrored manual in-person interventions, relying on one-way messaging to deliver curriculum content at regular intervals. Even in self-directed app environments, the self-directed component translated largely into re-visiting previous sessions or a content library at parents’ discretion [22]. The most interactive parts of such programs were help or ask-an-expert functions, where parents could reach out (to a real person) for specific advice [20, 27]. While these interventions could be valuable, it remains questionable whether a passive, largely informational approach is enough to lead to meaningful behavioral change. Additionally, this approach does not fully capitalize on the true potential of more current digital solutions such as artificial intelligence, e.g., personalized content selection [31], content personalization [32], real-time monitoring and feedback, adaptive learning [33], intelligent engagement, virtual assistance, developmental risks, and opportunities detection. For example, using artificial intelligence could help personalize content delivery based on user preferences and behavior, enhancing engagement and relevance. Similarly, intelligent engagement could tailor notifications, reminders, and other forms of communication to the specific caregiver needs to keep them engaged. Incorporating more automated features, such as personalized messaging systems, can facilitate real-time interaction, foster social exchanges, and cultivate online communities.

Only in one study were mothers asked to freely choose content without a curriculum on an internet-based support program [20]. Unfortunately, the program was not well described, and while the authors found program impacts on maternal mental health and wellbeing, they did not assess parenting behaviors or ECD. Further, mothers who did not spend a minimum amount of weekly time logged on received reminders, which might have different effects outside of a research context.

Keeping users engaged is a challenging but crucial issue for all self-guided digital interventions [9, 11]. In digital parenting interventions in HIC completion rates from 7% [34] to 15% [35] have been reported. Strategies employed to bolster compliance, such as weekly reminders and re-contacting families for updated contact information [18–20], underscore the importance of proactive measures to maximize participant involvement. However, evidence of the effectiveness of these strategies remains mixed [30]. This variability prompts critical questions about the factors influencing compliance levels, including the user-friendliness of digital platforms, relevance of intervention content, and cultural adaptation. Understanding these dynamics is essential for optimizing the design and impact of digital interventions.

Importantly, while the use of such strategies might be a crucial component to ensure participants receive an adequate dosage of the digital intervention, it raises questions about the feasibility of achieving comparable results in a real-world setting where such supporting measures might not always be appropriate or available. Furthermore, infrastructure and resource barriers play a significant role in shaping engagement with digital interventions. Factors like unstable internet access, lack of devices, or limited technical support can disproportionately hinder compliance in under-resourced settings. Without addressing these underlying inequities, the potential impact of digital interventions may remain unevenly distributed, further perpetuating health disparities.

### The Impact of Digital Parenting Interventions

There is an established evidence base showing that in-person parenting interventions following an established curriculum improve ECD if well-executed [6]. Nine of the included studies implemented digitized versions of such interventions but only 3 of them assessed ECD outcomes [21, 23, 27]. While 2 studies found some parent-reported ECD impact [23, 27], the one study using a direct child assessment had major intervention compliance issues and did not find ECD effects [21]. Notably, the other 2 studies using direct child assessments were not general parenting interventions [6]: One intervention with a comparatively minor parenting component compared high-to low-intensity mental health treatment groups among mothers screened positive for depression [26]. The study found positive effects for maternal mental health and exclusive breastfeeding, but no gains in parenting skills. This suggests that the parenting component was not emphasized enough, and that improvements in maternal depression might need more time to translate into ECD impact. The other intervention was a parent-training addition to hospital-based speech therapy for children [22]. Even with high compliance, a 4-month parent training might not be enough to show effects on children above sessions with a speech therapist. Finally, no studies directly compared a digital versus an in-person parenting intervention on ECD or parenting outcomes.

Digital parenting interventions generally showed positive effects on parenting behaviors and knowledge, such as increased activity involvement and better communication. These interventions also tended to improve parental wellbeing, particularly by reducing depression. Although many studies reported various parent outcomes, with some non-significant findings, the successes suggest potential benefits for child outcomes as well, which may be revealed in long-term observational studies.

### Successful Digital Interventions

Most studies that found intervention effects either included an important in-person component in the intervention (mixed-delivery interventions) or were able to attach the digital intervention to in-person interactions. This included a follow-up to a previous in-person intervention [18, 19], embedding it in monthly visits to the hospital [22], or creating a community on Whatsapp [30]. Even the individualized logon reminders used in one study could be argued to create a social pressure similar to in-person interactions that would increase compliance [20]. One possible explanation for this is that digital interventions alone might not (yet) provide sufficient levels of community, support and personalized exchange to keep parents engaged compared to in-person interactions.

However, in our review, we have only found 1 intervention testing the added value of an in-person component versus a purely digital intervention [27]. The study showed child and mother impacts compared to control in both intervention arms. Interestingly, the added in-person component only marginally improved knowledge scores of the adolescent mothers in Kenya and generated no additional impact on the parent-reported child development outcome compared to the purely digital intervention group.

Several distinguishing intervention features could potentially explain the success of this Kenyan intervention: i.) The frequency of messages was high with 5–10 weekly messages and the 9 months intervention period was longer than other purely digital interventions, ii.) there was an interactive component, where participants could directly use the messaging platform to ask questions to experts, iii.) participants were adolescent mothers, who might be more open to receive support compared to older mothers and iv.) the intervention might have integrated easily with existing social media habits [27].

### Limitations

This study has limitations. In accordance with accepted standards for scoping reviews, we did not examine the methodological quality and risk of bias of the studies. Due to the limited availability of data, we also were not able to conduct a formal meta-analysis.

### Recommendations for Future Research

Concretely, based on these findings we recommend 5 main directions for future research:

1. Assessing the efficacy, cost-effectiveness, and best practices for digital parenting interventions, including measurements of child impact.
2. Exploring and comparing the potential of digital versus in-person parenting interventions is a highly important avenue of investigation that is to date completely absent from the literature.
3. Examining how different delivery methods affect intervention outcomes and the potential benefits of offering participants a choice in the digital versus in-person elements. Similarly, examining how different strategies improve compliance and take-up of interventions.
4. Addressing the current one-way digital information delivery by incorporating personalized, AI-integrated curricula that can automate engagement and utilize interactive features to foster online communities.
5. Exploring strategies for translating the application and adherence of these interventions from research settings to real-world practice, a notably under-discussed area in existing literature.

### Conclusion

In summary, results show that to date there is a severe lack of data on ECD outcomes (direct assessments or parent-reported) in the context of digital parenting interventions. There is some indication that updating existing in-person interventions into a digitally-supported or digitally-enhanced version holds promise to make such interventions more effective. However, interventions that integrate more current digital solutions, such as artificial intelligence, for personalization, online community-building or real-time exchanges, are still missing. Finally, current studies often fail to address crucial factors such as acceptance, adoption, and sustained use of the interventions, which are integral to understanding their impact on ECD. Overall, it is evident that despite their promise for large-scale dissemination, the effectiveness of digital parenting interventions remains uncertain due to a scarcity of data from rigorous, larger studies that include ECD outcomes.

Future work in the domain of digital interventions should thus prioritize tailored approaches that actively monitor and enhance engagement for optimal outcomes. Addressing these issues will be essential for realizing the full potential of digital interventions in achieving meaningful and lasting outcomes for families.
