# Supplementary material for: Parenting in the Digital Age: A Scoping Review of Digital Early Childhood Parenting Interventions in Low- and Middle-Income Countries (LMIC)
Source: Public Health Rev. 2025 Jan 21;45:1607651. doi: 10.3389/phrs.2024.1607651 (PMC11790347; doi:10.3389/phrs.2024.1607651)
Supplement: Supplementary file 3 [file Table3.docx]

S3 Appendix:

List of excluded studies with reason for exclusion

| **Authors** | **Title** | **Year** | **Country** | **Exclusion reason** |
| --- | --- | --- | --- | --- |
| Aakash Ganju, Srini Satyan, Vatsal Tanna, Sonia Rebecca Menezes | AI for Improving Children’s Health: A Community Case Study | 2021 | India | no child development or parent outcomes |
| Abimpaye M, Dusabe C, Nzabonimpa JP, et al. | Improving parenting practices and development for young children in Rwanda: Results from a randomized control trial. International Journal of Behavioral Development | 2020 | Rwanda | not a digital intervention |
| Acquah-Gyan E, Acheampong PR, Mohammed A, et al. | User experiences of a mobile phone-based health information and surveillance system (mHISS): A case of caregivers of children under-five in rural communities in Ghana | 2022 | Ghana | not a parenting intervention |
| Alam M, Hickie IB, Poulsen A, et al. | Parenting app to support socio-emotional and cognitive development in early childhood: iterative codesign learnings from nine low-income and middle-income countries. | 2023 | NA | no intervention study |
| Alethea Desrosiers, Carolyn Schafer Rebecca Esliker; et al. | mHealth-Supported Delivery of an Evidence-Based FamilyHome-Visiting Intervention in Sierra Leone: Protocol for a PilotRandomized Controlled Trial | 2021 | Sierra Leone | no child development or parenting outcome |
| Alvarenga P, Cerezo MA, Wiese E, et al. | Effects of a short video feedback intervention on enhancing maternal sensitivity and infant development in low-income families | 2020 | Brazil | not a digital intervention |
| Archana B Patel, Priyanka N Kuhite, Ashraful Alam, et al. | M‐SAKHI—Mobile health solutions to help community providers promote maternal and infant nutrition and health using a community‐based cluster randomized controlled trial in rural India: A study protocol | 2019 | India | no child development or parenting outcome |
| Areemit R, Lumbiganon P, Suphakunpinyo C, et al. | A Mobile App, KhunLook, to Support Thai Parents and Caregivers With Child Health Supervision: Development, Validation, and Acceptability Study | 2020 | Thailand | no child development or parent outcomes |
| Areemit R, Saengnipanthkul S, Sutra S, et al. | Effectiveness of a Mobile App (KhunLook) Versus the Maternal and Child Health Handbook on Thai Parents' Health Literacy, Accuracy of Health Assessments, and Convenience of Use: Randomized Controlled Trial. | 2023 | Thailand | no child development or parent outcomes |
| Baby S. Nayak, Leslie Edward Lewis, Binu Margaret, et al. | Randomized controlled trial on effectiveness of mHealth (mobile/smartphone) based Preterm Home Care Program on developmental outcomes of preterms: Study protocol | 2018 | India | no child development or parenting outcome |
| Barron P, Peter J, LeFevre A, et al. | Mobile health messaging service and helpdesk for South African mothers (MomConnect): history, successes and challenges. | 2018 | South Africa | no child development or parenting outcome |
| Basu S, Garg S, Kumar R, Shukla A. | The willingness for using mobile phone for health education among women caregivers of under 5 children in an urban resettlement colony in Delhi, India. | 2017 | India | not an intervention study |
| Bonifacio LP, Franzon ACA, Zaratini FS, et al. | PRENACEL partner - use of short message service (SMS) to encourage male involvement in prenatal care: a cluster randomized trial. | 2020 | Brazil | does not continue after birth |
| Ceballos F, Hernandez MA, Olivet F, Paz C. | Assessing the use of cell phones to monitor health and nutrition interventions: Evidence from rural Guatemala. | 2020 | Guatemala | not a parenting intervention |
| Choi H, Van Riper M | mHealth Family Adaptation Intervention for Families of Young Children with Down Syndrome: A Feasibility Study | 2020 | South Korea | not a LMIC |
| Chowdhury M, Shiblee S, Jones H | Does mHealth voice messaging work for improving knowledge and practice of maternal and newborn healthcare? | 2019 | Bangladesh | not a parenting intervention |
| Comer JS, Furr JM, Miguel EM, et al. | Remotely delivering real-time parent training to the home: An initial randomized trial of Internet-delivered parent-child interaction therapy (I-PCIT). | 2017 | USA | not a LMIC |
| Crouse JJ, LaMonica HM, Song YJC, et al. | Designing an App for Parents and Caregivers to Promote Cognitive and Socioemotional Development and Well-being Among Children Aged 0 to 5 Years in Diverse Cultural Settings: Scientific Framework. | 2023 | NA | not a parenting intervention |
| Daniel Fatori, Adriana Argeu, Helena Brentani; et al. | Maternal Parenting Electronic Diary in the Context of a Home Visit Intervention for Adolescent Mothers in an Urban Deprived Area of São Paulo, Brazil: Randomized Controlled Trial | 2020 | Brazil | not a digital intervention |
| Doan TTD, Tran TC, Pham NM, et al. | Designing and developing a mobile app (BeBo) in a randomized controlled trial study to promote breastfeeding among Vietnamese mothers. | 2023 | Vietnam | not a parenting intervention |
| Escobedo L, Arriaga R. | Understanding the challenges of deploying a milestone-tracking application in a cross-cultural context. | 2022 | USA | not a LMIC |
| Farzaneh Rezaie, Mohammadreza Firouzkouhi, Abdolghani Abdollahimohammad, Mahin Naderifar | Comparing the effect of education through SMS with face-to-face method on awareness and care in mothers with premature neonates | 2019 | Iran | not a parenting intervention |
| Gilkerson J, Richards JA, Topping K. | Evaluation of a LENA-Based Online Intervention for Parents of Young Children. Journal of Early Intervention | 2017 | USA | not a LMIC |
| Guo L, Zhang J, Mu L, Ye Z. | Preventing postpartum depression with mindful self-compassion intervention: a randomized control study. | 2020 | China | not a parenting intervention |
| Harpreet Singh, Raghuram Mallaiah, Gautam Yadav, et al. | iCHRCloud: Web & Mobile based Child Health Imprints for Smart Healthcare | 2017 | India | not a parenting intervention |
| Hazra A, Khan ME, Mondal SK, et al. | Mobile phone messaging to husbands to improve maternal and child health behavior in India. | 2018 | India | not a parenting intervention |
| Jannati N, Mazhari S, Ahmadian L, Mirzaee M. | Effectiveness of an app-based cognitive behavioral therapy program for postpartum depression in primary care: A randomized controlled trial. | 2020 | Iran | not a parenting intervention |
| Khader Y, Maalouf W, Abu Khdair M, et al. | Scaling the Children Immunization App (CIMA) to Support Child Refugees and Parents in the Time of the COVID-19 Pandemic: A Social Capital Approach to Scale a Smartphone Application in Zaatari Camp, Jordan. | 2022 | Jordan | no child development or parenting outcome |
| Kitsao-Wekulo P, Langat N, Nampijja M, et al. | Development and feasibility testing of a mobile phone application to track children’s developmental progression | 2021 | Kenya | no child development or parent outcomes |
| Kocak V, Ege E, Iyisoy MS. | The development of the postpartum mobile support application and the effect of the application on mothers’ anxiety and depression symptoms. | 2021 | Turkey | is not a parenting intervention. secondary outcome fits into our criteria |
| Kola L, Abiona D, Oladeji B, et al. | Theory-driven development of a mobile phone supported intervention for adolescents with perinatal depression | 2022 | Nigeria | not an intervention study |
| Krishna D, Muthukaruppan S, Bharathwaj A, et al. | Rapid-Cycle Evaluation in an Early Intervention Program for Children With Developmental Disabilities in South India: Optimizing Service Providers’ Quality of Work-Life, Family Program Engagement, and School Enrollment. | 2020 | India | no child development or parenting outcome |
| Lee K, Chang I, Wu T, Chen R. | The Moderating Role of Perceived Interactivity in the Relationship Between Online Customer Experience and Behavioral Intentions to Use Parenting Apps for Taiwanese Preschool Parents. | 2022 | Taiwan | not a LMIC |
| LeFevre A, Dane P, Copley C, et al. | Unpacking the performance of a mobile health information messaging program for mothers (MomConnect) in South Africa: evidence on program reach and messaging exposure. | 2018 | South Africa | not an intervention study |
| Li X, Zhang Y, Ye Z, Huang L, Zheng X. | Development of a Mobile Application of Internet-Based Support Program on Parenting Outcomes for Primiparous Women | 2021 | China | no child development or parent outcomes |
| Lisa B. Sheeber, Edward G. Feil, John R. Seeley, et al. | Mom-Net: Evaluation of an Internet-Facilitated Cognitive Behavioral Intervention for Low-Income Depressed Mothers | 2017 | USA (not specified but inferred) | not a LMIC |
| Lucy McGoron, Erica Hvizdos, Erika L. Bocknek, et al. | Feasibility of Internet-based Parent Training for Low-income Parents of Young Children | 2018 | USA | not a LMIC |
| Misago N, Habonimana D, Ciza R, et al. | A digitalized program to improve antenatal health care in a rural setting in North-Western Burundi: Early evidence-based lessons. | 2023 | Burundi | not a parenting intervention |
| Munirul M Haque; Masud Rabbani; Dipranjan Das Dipa; et al. | Informing Developmental Milestone Achievement for Children With Autism: Machine Learning Approach | 2021 | Bangladesh | not a parenting intervention |
| Ngai FW, Chan PS, Bandura, et al. | A qualitative evaluation of telephone-based cognitive-behavioral therapy for postpartum mothers. | 2019 | Hong Kong | not a LMIC |
| Ngai FW, Wong PWC, Chung KF, et al. | The effect of telephone-based cognitive-behavioural therapy on parenting stress: A randomised controlled trial. | 2016 | Hong Kong | not a LMIC |
| Ngai FW, Wong PWC, Chung KF, et al. | Randomized controlled trial of telephone-based cognitive-behavioral therapy on parenting self-efficacy and satisfaction. | 2019 | Hong Kong | not a LMIC |
| Obasola O, Mabawonku I. | Women’s Use of Information and Communication Technology in Accessing Maternal and Child Health Information in Nigeria. | 2017 | Nigeria | not an intervention study |
| Parker R, Dmitrieva E, Frolov S, Gazmararian J. | Text4baby in the United States and Russia: An Opportunity for Understanding How mHealth Affects Maternal and Child Health. | 2012 | USA | not a LMIC |
| Patricia Kitsao-Wekulo,Nelson Kipkoech Langat,Margaret Nampijja, Elizabeth Mwaniki, Kenneth Okelo, Elizabeth Kimani-Murage | Development and feasibility testing of a mobile phone application to track children’s developmental progression | 2021 | Kenya | no child development or parent outcomes |
| Paul JJ, Dardar S, River LM, et al. | Telehealth adaptation of perinatal mental health mother-infant group programming for the COVID-19 pandemic | 2022 | USA | not a LMIC |
| Peiris DR, Wijesinghe MSD, Gunawardana BMI, et al. | Mobile Phone-Based Nutrition Education Targeting Pregnant and Nursing Mothers in Sri Lanka. | 2023 | Sri Lanka | not a parenting intervention |
| Phagdol T, Nayak BS, Lewis LE, et al. | Effectiveness of mHealth application in improving knowledge of mothers on preterm home care | 2022 | India | not a parenting intervention |
| Poulsen A, Hickie IB, Alam M, LaMonica HM | User Experience Co-Design of a Mobile Application to Support Childrearing in Low- and Middle-Income Countries. | 2023 | NA | not an intervention study |
| Prieto J, Zuleta C, Rodriguez J. | Modeling and testing maternal and newborn care mHealth interventions: a pilot impact evaluation and follow-up qualitative study in Guatemala. | 2017 | Guatemala | not a parenting intervention |
| Dias RD, Silva KC, Lima MR, et al. | A Mobile Early Stimulation Program to Support Children with Developmental Delays in Brazil | 2018 | Brazil | no child development or parent outcomes |
| Roben CKP, Kipp E, Schein SS, et al. | Transitioning to telehealth due to COVID-19: Maintaining model fidelity in a home visiting program for parents of vulnerable infants. | 2022 | USA | not a LMIC |
| Rosa Sze Man Wong, Esther Yee Tak Yu, Thomson Wai-Lung Wong, et al. | Development and pilot evaluation of a mobile app on parent-child exercises to improve physical activity and psychosocial outcomes of Hong Kong Chinese children | 2020 | China | children too old |
| Rotheram-Fuller E, Swendeman D, Becker K, et al. | Adapting Current Strategies to Implement Evidence-Based Prevention Programs for Paraprofessional Home Visiting | 2017 | USA | not a LMIC |
| Sajedi F, Habibi E, Shahshahanipour S, et al. | An Approach towards Promoting Iranian Caregivers’ Knowledge on Early Childhood Development. | 2018 | Iran | not a parenting intervention |
| Sedigheh Khanjari, Edward F. Bell, Leila Alsadat Sadeghi, et al., | The impact of a mobile health intervention on the sense of coherence and quality of life of mothers with premature infants | 2021 | Iran | not a parenting intervention |
| Seshu U, Khan HA, Bhardwaj M, et al. | A qualitative study on the use of mobile-based intervention for perinatal depression among perinatal mothers in rural Bihar, India. | 2021 | India | not a parenting intervention |
| Shefaly Shorey, Yvonne Peng, Mei Ng, et al. i | Effectiveness of a Technology-Based Supportive Educational Parenting Program on Parental Outcomes (Part 1): Randomized Controlled Trial | 2019 | Singapore | not a LMIC |
| Sieverson C, Santelices MP, Farkas C, et al. | Effects of a mentalization-based group intervention with videofeedback for mothers of preschool children | 2021 | Chile | not a digital intervention |
| Solange Parra, Alejandra Ortega, Rebecca Kanter, et al. | Process of developing text messages on healthy eating and physical activity for Chilean mothers with overweight or obese preschool children to be delivered via WhatsApp | 2018 | Chile | no child development or parenting outcome |
| Solis-Cordero K, Marinho P, Camargo P, et al. | The BEM Program: An innovative online parenting program for socioeconomically disadvantaged caregiver-child dyads in Brazil. | 2023 | Brazil | no child development or parenting outcome |
| Sungji Ha, Jung Hwa Han, Jaeun Ahn, et al. | Pilot study of a mobile application-based intervention to induce changes in neural activity in the frontal region and behaviors in children with attention deficit hyperactivity disorder and/or intellectual disability | 2022 | South Korea | not a LMIC |
| Tenzin Phagdol, Baby S Nayak, Leslie Edward Lewis, et al. | Designing a mobile health intervention for preterm home care: Application of conceptual framework | 2022 | India | no child development or parent outcomes |
| Thobias J, Kiwanuka A. | Design and implementation of an m-health data model for improving health information access for reproductive and child health services in low resource settings using a participatory action research approach. | 2018 | Tanzania | not a parenting intervention |
| Wenxiao Zhao Shaoni Guo | STEM in English for Early Childhood Ecological Awareness in China | 2019 | China | no child development or parenting outcome |
| Young M, Baik D, Reinsma K, et al. | Evaluation of mobile phone-based Positive Deviance/Hearth child undernutrition program in Cambodia | 2021 | Cambodia | not a parenting intervention |
